# Retraction: Biofabrication of streptomycin-conjugated calcium phosphate nanoparticles using red ginseng extract and investigation of their antibacterial potential

**DOI:** 10.1371/journal.pone.0228300

**Published:** 2020-01-21

**Authors:** The PLOS ONE Editors

After this article [1] was published, it came to light that there was a potential competing interest between the handling Academic Editor and one or more authors, who appear as co-authors on recent publications. We had the article re-assessed by a different member of *PLOS ONE*’s Editorial Board who advised that there are a number of concerns and that overall the article does not meet *PLOS ONE*’s publication criteria.

The following concerns were raised in the post-publication editorial assessment:

- The nanoparticles (NPs) were not sufficiently characterized to meet community standards. They should have been characterized by a variety of methods to demonstrate zeta potential, population distribution, and polydispersity index. Furthermore, the reported electron microscopy data show irregular morphology and agglomeration, and NP size details are not provided.
- The data reported in the article do not adequately support claims about streptomycin conjugation to NPs or verify how much streptomycin was linked to the NPs.
- The study relied on “zone of inhibition” (diffusion-based) assays to examine antibacterial activity. Per community standards, antibacterial activity and minimum inhibitory concentration (MIC) results need to be determined in liquid culture experiments; data from “zone of inhibition” assays do not adequately support the reported results and conclusions.
- The article’s figures do not include control data for CPG NPs (calcium phosphate nanoparticles synthesized using red ginseng extract) as a comparator for CPG-S, or streptomycin-linked CPG NPs, for example, in reporting Fourier-transform infrared spectroscopy, X-ray powder diffraction, thermogravimetric and differential thermogravimetric analysis spectra and zone of inhibition results.
- Conclusions about the mode of action were based on scanning electron microcopy image data, this methodology does not provide mechanistic insight and thus conclusions about the mode of action were not adequately supported.
- The statistical analyses and results were not adequately reported, and it is unclear from the article which groups were compared in the analyses. The authors clarified that they used ANOVA and Duncan’s test to compare antibacterial activity between pathogens and samples.
- The relevance of the study to dental care was called into question in light of the high MICs reported, lack of data to demonstrate activity of NPs against oral pathogens, and known side effects and bacterial resistance associated with streptomycin.

The authors offered to perform additional experiments to address some of the items outlined. However, the extent of the issues is such that concerns about study design and experimental rigor would remain which call into question the validity and reproducibility of the results and conclusions reported in the article.

In light of the above concerns, the *PLOS ONE* Editors retract this article.

The authors did not agree with the retraction.
